# Synthesis of New *C*- and *N*-β-d-Glucopyranosyl Derivatives of Imidazole, 1,2,3-Triazole and Tetrazole, and Their Evaluation as Inhibitors of Glycogen Phosphorylase

**DOI:** 10.3390/molecules23030666

**Published:** 2018-03-15

**Authors:** Sándor Kun, Éva Bokor, Ádám Sipos, Tibor Docsa, László Somsák

**Affiliations:** 1 Department of Organic Chemistry, University of Debrecen, P.O. Box 400, H-4002 Debrecen, Hungary; kun.sandor@science.unideb.hu (S.K.); bokor.eva@science.unideb.hu (É.B.); 2 Department of Medical Chemistry, Faculty of Medicine, University of Debrecen, Egyetem tér 1, H-4032 Debrecen, Hungary; sipos.adam@med.unideb.hu (Á.S.); tdocsa@med.unideb.hu (T.D.)

**Keywords:** *C*-glucosyl heterocycle, *N*-glucosyl heterocycle, 1,2,3-triazole, imidazole, tetrazole, glycogen phosphorylase, inhibitor, structure-activity relationship

## Abstract

The aim of the present study was to broaden the structure-activity relationships of *C*- and *N*-β-d-glucopyranosyl azole type inhibitors of glycogen phosphorylase. 1-Aryl-4-β-d-gluco-pyranosyl-1,2,3-triazoles were prepared by copper catalyzed azide-alkyne cycloadditions between *O*-perbenzylated or *O*-peracetylated β-d-glucopyranosyl ethynes and aryl azides. 1-β-d-Gluco-pyranosyl-4-phenyl imidazole was obtained in a glycosylation of 4(5)-phenylimidazole with *O*-peracetylated α-d-glucopyranosyl bromide. *C*-β-d-Glucopyranosyl-*N*-substituted-tetrazoles were synthesized by alkylation/arylation of *O*-perbenzoylated 5-β-d-glucopyranosyl-tetrazole or from a 2,6-anhydroheptose tosylhydrazone and arenediazonium salts. 5-Substituted tetrazoles were glycosylated by *O*-peracetylated α-d-glucopyranosyl bromide to give *N*-β-d-glucopyranosyl-*C*-substituted-tetrazoles. Standard deprotections gave test compounds which were assayed against rabbit muscle glycogen phosphorylase *b*. Most of the compounds proved inactive, the best inhibitor was 2-β-d-glucopyranosyl-5-phenyltetrazole (IC_50_ 600 μM). These studies extended the structure-activity relationships of β-d-glucopyranosyl azole type inhibitors and revealed the extreme sensitivity of such type of inhibitors towards the structure of the azole moiety.

## 1. Introduction

Glycogen phosphorylase inhibitors (GPIs) attract considerable scientific interest [1,2,3] since such compounds may be applied in finding new therapies against type two diabetes [4,5,6], myocardial [7,8] and cerebral [9,10] ischemias, and tumors [11,12,13,14,15,16]. In addition to demonstrating in vivo antihyperglycemic activities [17,18,19,20,21], physiological studies with several GPIs revealed unprecedented effects on hepatic metabolism [22] and improvement of pancreatic β-cell function [23].

A large variety of compounds have been designed and synthesized for the inhibition of GP [24,25]. Among them different derivatives of d-glucose represent the largest class of inhibitors. Within glucose-derived compounds *N*-acyl-β-d-glucopyranosylamines (**I** in Table 1) [26,27,28,29] were among the first low micromolar inhibitors of rabbit muscle GP *b* (RMGP*b*), the prototype of these enzymes [30]. Based on the concept of bioisosterism [31,32,33] many sorts of *N*- and *C*-β-d-gluco-pyranosyl heterocycles, wherein the hetero-ring replaced the NHCO moiety of **I** as its bioisostere, were synthesized and tested against RMGP*b*. Thus, *N*-β-d-glucopyranosyl 1,2,3-triazoles **II** [29,34] had comparable inhibitory effects to those of amides **I**. Among *C*-β-d-glucopyranosyl derivatives three isomeric oxadiazoles **III**–**V** were studied to show that the constitution of the heterocycle had a decisive bearing on the efficiency. Thus, 1,3,4-oxadiazoles **III** [35,36,37] proved to be rather weak inhibitors, the 3-glucosyl-5-substituted isomers **IV** [38] were somewhat more efficient (especially the 2-naphthyl derivative **IVc**) and the 5-glucosyl-3-substituted counterparts **V** [37,39] showed low micromolar inhibition. *C*-Glucosyl 1,2,4-triazoles **VI** [40,41] and imidazoles **VII** [42,43] featuring hydrogen bond donor heterocycles were significantly more effective. 

Actually, these compounds are the best glucose analogue inhibitors of GP known to date. Their efficiency, among other factors, is due to the formation of a H-bridge between the heterocycle and the His-377 main chain carbonyl group in the active site of the enzyme. 5-Glucosyltetrazole **VIII** [35], although capable of H-bond formation, proved inactive. It is remarkable that, besides the properties of the heterocycle, also the nature and the size of the substituent of the azole moiety had a very significant influence on the activity of the inhibitors. While methyl substituted derivatives in column **a** were practically inactive, a phenyl appendage (column **b**) made much more efficient compounds and the 2-naphthyl derivatives (column **c**) proved to be the strongest inhibitors. With these preliminaries in mind, in order to make the structure-activity relationship of *N*- and *C*-β-d-glucopyranosyl azole type compounds more complete, we envisaged to synthesize the “missing” counterparts of the above glucose derivatives. In this paper the syntheses and enzymatic evaluation of 4-β-d-glucopyranosyl-1-substituted-1,2,3-triazoles **IX**, 1-β-d-glucopyranosyl-4-substituted imidazoles **X**, 5-β-d-glucopyranosyl-2-substituted tetrazoles **XI**, and 2-β-d-glucopyranosyl-5-substituted tetrazoles **XII** are presented.

## 2. Results and Discussion

For the syntheses of *C*-glucosyl 1,2,3-triazoles of type **IX** several methods were published and this chemistry was reviewed last year [44]. Our work, summarized in Table 2, started with *O*-perbenzylated *C*-glucosyl acetylene **1** described in the literature [45]. Copper catalyzed azide-alkyne cycloaddition (CuAAC) [46] was effected from **1** either by pre-formed aryl azides with CuO(CO)C_3_H_7_(PPh_3_)_2_ as the catalyst [47] (method *a*) or azides obtained in situ from areneboronic acids [48,49] (method *b*) to give 1,2,3-triazoles **2a–c** in very good yields. Removal of the *O*-benzyl protecting groups from **2a** by usual catalytic hydrogenation (method *c*) gave excellent yield of **5a**, however, under the same conditions **2c** gave an inseparable product mixture. After *O*-peracetylation (method *e*) of the mixture the products could be separated and identified as **4c** and a partially saturated derivative **4d**. Since the formation of a tetrahydronaphthyl by-product under catalytic hydrogenation was observed previously with a 2-naphthyl substituted *C*-glucopyranosyl 1,2,4-triazole [50] hydrogenolytic deprotection of **2b** was not attempted. Instead, the protecting groups were exchanged to acetate esters as reported to get *O*-peracetylated acetylene **3 [51]**. CuAAC from **3** produced triazoles **4b** and **4c** in very good yields. Formation of **4b** was also effected from **2b** by a direct exchange of protective groups by method *d* [51]. Removal of the *O*-acetyl groups from **4b,c** under Zemplén conditions (method *f*) gave the targeted **5b**,**c** in excellent yields.

**Scheme 1:**
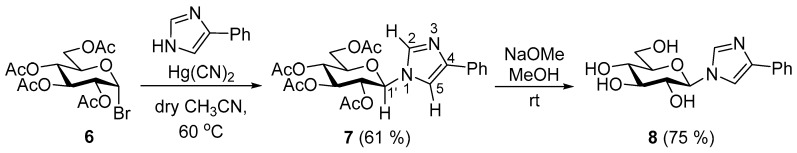
Synthesis of 1-(β-d-glucopyranosyl)-4-phenyl-imidazole.

For the preparation of an imidazole of type **X** a literature method [52] was adapted. Thus, acetobromoglucose **6** was reacted with 4-phenyl-imidazole in the presence of Hg(CN)_2_ in acetonitrile to give 1-glucopyranosyl-4-phenyl-imidazole **7** (Scheme 1). Due to the tautomerism of imidazoles the formation of the isomeric 1-glucopyranosyl-5-phenyl-imidazole would also be possible, however, this was excluded on the basis of a HMBC measurement. Specifically, the observation of cross peaks between H-1’–C-2, H-1’‒C-5, C-1’‒H-2, and C-1’‒H-5 clearly indicated the formation of **7**. *O*-Deacetylation of **7** by the Zemplén method gave **8** in good yield.

Next we turned to the synthesis of *C*-glucopyranosyl tetrazoles of type **XI**. While 5-(β-d-gluco-pyranosyl)tetrazoles (e.g., **9**) are long known compounds ([35,49] and references cited therein), no *N*-substituted derivatives could be located in the literature. For the preparation of the phenyl substituted derivatives a copper catalyzed reaction [53] of **9** and benzeneboronic acid was applied (Table 3, conditions *a*). Although the tautomerism of the tetrazole moiety could have facilitated the formation of regioisomers, only **10a** was obtained in excellent yield as it was claimed in the cited paper. By modifying a literature procedure [54], compound **10a** was also prepared, albeit in lower yield, from tosylhydrazone **12** [55,56] and benzenediazonium tetrafluoroborate [57,58] (conditions *c*). For the methylation of **9** a method [59] applied for the synthesis of *C*-glycofuranosyl tetrazoles was adapted. Thus, **9** was reacted with diazomethane to give a 1:1 mixture of the regioisomeric tetrazoles **10e** and **11e** in very good overall yield (conditions *b*). Removal of the ester protecting groups by the Zemplén protocol gave the test compounds **13** and **14** in very good yields (conditions *d*). The regioisomers of the formed *C,N*-disubstituted tetrazoles could easily be identified by the ^13^C-NMR signal of the C-5 carbons. It is well known that the tetrazole carbon of 2,5-disubstituted derivatives (162–167 ppm) is shifted downfield by ~10 ppm in comparison to that of the 1,5-disubstituted counterparts (152–156 ppm) [60], and this is clearly visible in the obtained data shown in Table 3. In addition, for **11e** ^1^H-^1^H NOEs were observed between the CH_3_ protons and the pyranose H-1’ and H-2’, while for **10e** the NOE spectrum did not indicate proximity between the substituents of the tetrazole.

For the synthesis of *N*-(β-d-glucopyranosyl)-5-substituted-tetrazoles a literature protocol was applied to give **15a** and **16a** [61] in the reaction of acetobromoglucose **6** and 5-phenyltetrazole [62] (Table 4). From a similar transformation of **6** with 5-methyltetrazole [62] only the 2,5-disubstituted **15e** could be isolated in moderate yield and the formation of the HBr elimination product 2-acetoxy-d-glucal **17** was observed in a significant amount. Protecting group removal was effected by the Zemplén method to furnish the test compounds **18** and **19** in very good yields. The regioisomeric tetrazoles **15**, **18** vs. **16**, **19** were identified on the basis of the C-5 chemical shifts as described above (see respective data in Table 4). 

The new compounds were assayed against rabbit muscle glycogen phosphorylase *b* enzyme (RMGP*b*) as described earlier [27] and the results are collected in Table 5. The inefficiency of *C*-glucopyranosyl 1,2,3-triazoles **5** (entries 4–6) as compared to the micromolar inhibition of the *N*-glucopyranosyl counterparts **II** in Table 2 came as a surprise, since the size of the heterocycle and the position of the H-bond donor and acceptor sites of the ring must not have been altered by the interchange of the substituents. A comparison of the inhibitory efficiency of *N*-benzoyl-β-d-gluco-pyranosylamine **Ib** (entry 1) with its “reversed” counterpart *N*-phenyl-2,6-anhydro-d-*glycero*-d-*gulo*-heptonamide **20** (shown in entry 3) results in a ratio of 38–67 (~53 as an average). Multiplication of the inhibition constants of **IIb** (entry 2) with this average factor to predict the efficiency of **5a** (entry 4) gives values of ~7900–8500 μM, a range being well beyond the concentrations investigated in this study (max 625 μM). Nevertheless, our observations may refer to a strong directionality in the amide-1,2,3-triazole bioisosterism (mostly ignored in related studies [63,64]) indicating that the proper replacement must correspond to the pairs **Ib–IIb** and **20–5a** in entries 1–2 and 3–4, respectively.

*N*-Glucosylimidazole **8** (entry 7) as well as *N*-substituted-5-glucopyranosyltetrazoles **13** and **14** (entries 8–10) proved non-inhibitory in the investigated concentration range. From the *N*-glucopyranosyl-5-substituted tetrazoles **18** and **19** (entries 11–13) only the 5-phenyl derivative **18a** showed very weak inhibition (entry 11). This study has corroborated that the inhibition of glycogen phosphorylase by *N*- and *C*-glucopyranosyl azole type compounds is extremely sensitive to the properties of the heterocycle. 

## 3. Experimental

### 3.1. General Methods

Anhydrous solvents were prepared by standard methods. CH_2_Cl_2_, CHCl_3_ and EtOAc were distilled from P_4_O_10_ and stored over 4 Å molecular sieves. MeOH was distilled over Mg turnings and iodine. Acetone was dried by distillation from CaSO_4_. Anhydrous pyridine (VWR, Vienna, Austia) and EtOH (Molar Chemicals, Halásztelek, Hungary) were used as received. Melting points were measured on a Kofler hot stage and are uncorrected. Optical rotations were determined on a P-2000 polarimeter (Jasco, Easton, MD, USA) at room temperature. NMR spectra were recorded with DRX360 (360/90 MHz for ^1^H/^13^C) and DRX400 (400/100 MHz for ^1^H/^13^C) spectrometers (Bruker, Karlsruhe, Germany). Chemical shifts are referenced to internal Me_4_Si (^1^H) or the residual solvent signal (^13^C). HRMS spectra were recorded with a Bruker maXis II spectrometer with electrospray ionization technique. TLC was performed on DC Alurolle Kieselgel 60 F_254_ (Merck, Darmstadt, Germany), the plates were visualized by gentle heating. For column chromatography Kieselgel 60, 63–200 µm (Molar Chemicals) was used. Organic solutions were dried over anhydrous MgSO_4_ and concentrated in vacuo at 40–50 °C (water bath). Alkynes **1** [45] and **3** [51], tetrazole **9** [49], tosylhydrazone **12** [55,56], 5-phenyltetrazole [62], 5-methyltetrazole [62], and benzenediazonium tetrafluoroborate [57,58] were prepared according to literature procedures.

#### 3.1.1. General Procedure 1 for the Synthesis of O-Peracetylated or O-Perbenzylated 1-aryl-4-β-d-Glucopyranosyl-1,2,3-triazoles from Azido-Arenes

To the solution of the corresponding alkyne (**1** or **3**) in anhydr. CH_2_Cl_2_ (0.1 mmol/mL) the azido-arene and CuO(CO)C_3_H_7_(PPh_3_)_2_ were added and the mixture was stirred at rt for the given time (20 min–5 h) while the reaction was monitored by TLC (eluent: hexane-EtOAc 4:1 for *O*-benzylated cmpounds, 1:1 for *O*-acetylated compounds). After total consumption of the alkyne the solvent was evaporated and the residue purified by column chromatography.

#### 3.1.2. General Procedure 2 for the Synthesis of O-Peracetylated or O-Perbenzylated 1-aryl-4-β-d-Glucopyranosyl-1,2,3-triazoles from Arylboronic Acids by Using CuSO_4_/L-Ascorbic Acid Catalytic System

Arylboronic acid (1 equiv.) was dissolved in MeOH (5 mL/mmol), NaN_3_ (1.2 equiv.) and CuSO_4_·5H_2_O (0.1 equiv.) were added and the reaction mixture was stirred at r.t. After 18 h distilled water (10 mL/mmol), CH_2_Cl_2_ (10 mL/mmol), the corresponding alkyne (**1** or **3**, 0.3 equiv.) and L-ascorbic acid (0.5 equiv.) was added and the mixture was stirred at 50 °C (oil bath temp.). When TLC showed complete disappearance of the alkyne (eluent: hexane-EtOAc 4:1 for *O*-benzylated compounds, 1:1 for *O*-acetylated compounds) the mixture was diluted with CH_2_Cl_2_ and water. After separation of the phases the aqueous layer was washed with CH_2_Cl_2_. The combined organic phases were dried, concentrated under reduced pressure and chromatographed to yield the pure 1,2,3-triazole.

#### 3.1.3. General Procedure 3 for Removal of the O-Acetyl Protecting Groups

An *O*-acyl protected compound (100 mg) was dissolved in anhydr. MeOH (5 mL), a few drops of ~1 M solution of NaOMe/MeOH was added and the mixture was left to stand at r.t. After complete conversion (TLC monitoring, CHCl_3_-MeOH 7:3) the reaction mixture was neutralized with Amberlyst 15 (hydrogen form). After removal of the resin by filtration, the solvent was evaporated in vacuo and the crude product was purified by column chromatography (CHCl_3_-MeOH 9:1).

#### 3.1.4. General Procedure 4 for the Synthesis of O-Peracetylated *N*-(β-d-Glucopyranosyl)tetrazoles

Freshly flame dried K_2_CO_3_ (10 equiv., 1.68 g, 12.2 mmol), 4 Å powdered molecular sieves (500 mg), 2,3,4,6-tetra-*O*-acetyl-α-d-glucopyranosyl bromide (**6**, 500 mg, 1.22 mmol) and a 5-substituted tetrazole (2 equiv., 2.44 mmol) were mixed in a round bottom flask, anhydr. acetone (15 mL) was added and the mixture was stirred and refluxed for 8 h. After removal of the solids by filtration the filtrate was concentrated under reduced pressure and chromatographed to give *N*-(β-d-glucopyranosyl)tetrazoles.

### 3.2. Characterization of the Comounds

*1-Phenyl-4-(2′,3′,4′,6′-tetra-O-benzyl-β-d-glucopyranosyl)-1H-1,2,3-triazole* (**2a**). Prepared according to general procedure 1 from alkyne **1** (153 mg, 0.28 mmol), azidobenzene (33 mg, 0.28 mmol) and CuO(CO)C_3_H_7_(PPh_3_)_2_ (2 mg, 0.003 mmol). Reaction time: 20 min. Purified by column chromatography (eluent: hexane-EtOAc = 4:1 → 2:1 gradient) to yield 145 mg (78%) white crystals. R_f_ = 0.56 (hexane-EtOAc = 2:1); Mp: 160–162 °C; [α]_D_ = −16 (c 0.53, CHCl_3_); ^1^H-NMR (CDCl_3_, 360 MHz) δ (ppm): 7.81 (1H, s, triazole H-5), 7.66–6.99 (25H, m, Ar), 4.98, 4.93 (2 × 1H, 2 d, *J* = 11.1 Hz, PhC*H*_2_), 4.88, 4.60 (2 × 1H, 2 d, *J* = 10.7 Hz, PhC*H*_2_), 4.69, 4.40 (2 × 1H, 2d, *J* = 10.9 Hz, PhC*H*_2_), 4.57, 4.52 (2 × 1H, 2d, *J* = 12.1 Hz, PhC*H*_2_), 4.61 (1H, d, *J* = 9.6 Hz, H-1’), 3.98, 3.86 (2H, 2 pseudo t, *J* = 9.4, 8.8 Hz, H-2’ and/or H-3’ and/or H-4’), 3.79–3.70 (3H, m, H-2’ or H-3’ or H-4’, H-6’a, H-6’b), 3.66 (1H, ddd, *J* = 9.4, 3.5, 2.4 Hz, H-5’); ^13^C-NMR (CDCl_3_, 90 MHz) δ (ppm): 146.4 (C-4), 138.5–136.9 (Ar), 129.6–127.5 (Ar), 120.9 (triazole C-5), 120.4 (Ar), 86.9, 81.5, 79.4, 78.1, 74.0 (C-1’–C-5’), 75.5, 75.0, 74.7, 73.4 (4 × Ph*C*H_2_), 69.0 (C-6’). ESI-HRMS positive mode (*m*/*z*): calcd. for C_42_H_42_N_3_O_5_^+^ ([M + H]^+^): 668.3119. Found: 668.3116.

*1-(Naphthalen-2-yl)-4-(2′,3′,4′,6′-tetra-O-benzyl-β-d-glucopyranosyl)-1H-1,2,3-triazole* (**2b**). Prepared according to general procedure 2 from 2-naphthylboronic acid (52 mg, 0.30 mmol), CuSO_4_·5H_2_O (8 mg, 0.03 mmol), NaN_3_ (24 mg, 0.36 mmol), L-ascorbic acid (27 mg, 0.15 mmol) and alkyne **2** (50 mg, 0.09 mmol). Reaction time: 1.5 h. Purified by column chromaography (EtOAc-hexane 1:7 → 1:6 gradient) to yield 52 mg (79%) white crystalline product. R_f_ = 0.23 (hexane-EtOAc = 4:1); Op: 140–141 °C; [α]_D_ = −19 (c 0.52, CHCl_3_); ^1^H-NMR (CDCl_3_, 360 MHz) δ (ppm): 8.07–7.81 (5H, m, Ar), 7.92 (1H, s, triazole H-5), 7.59–7.53 (2H, m, Ar), 7.38–7.01 (20H, m, Ar), 4.99, 4.95 (2 × 1H, 2 d, *J* = 11.1 Hz, PhC*H*_2_), 4.88, 4.61 (2 × 1H, 2 d, *J* = 10.7 Hz, PhC*H*_2_), 4.71, 4.44 (2 × 1H, 2 d, *J* = 10.9 Hz, PhC*H*_2_), 4.59, 4.53 (2 × 1H, 2 d, *J* = 12.2 Hz, PhC*H*_2_), 4.65 (1H, d, *J* = 9.7 Hz, H-1’), 3.99, 3.88 (2H, 2 pseudo t, *J* = 9.4, 8.8 Hz, H-2’ and/or H-3’ and/or H-4’), 3.81–3.72 (3H, m, H-2’ or H-3’ or H-4’, H-6’a, H-6’b), 3.68 (1H, ddd, *J* = 9.4, 3.5, 1.3 Hz, H-5’); ^13^C-NMR (CDCl_3_, 90 MHz) δ (ppm): 146.4 (triazole C-4), 138.5–132.8 (Ar), 129.9–126.9 (Ar), 121.1 (triazole C-5), 118.9, 118.4 (Ar), 87.0, 81.4, 79.5, 78.2, 74.1 (C-1’–C-5’), 75.6, 75.1, 74.7, 73.4 (4 × Ph*C*H_2_), 69.1 (C-6’). ESI-HRMS positive mode (*m*/*z*): calcd. for C_46_H_44_N_3_O_5_^+^ ([M + H]^+^): 718.3275. Found: 718.3273.

*1-(Naphthalen-1-yl)-4-(2’,3’,4’,6’-tetra-O-benzyl-β-d-glucopyranosyl)-1H-1,2,3-triazole* (**2c**). Prepared according to General procedure 1 from alkyne **2** (150 mg, 0.27 mmol), 1-azidonaphthalene (46 mg, 0.27 mmol) and CuO(CO)C_3_H_7_(PPh_3_)_2_ (2 mg, 0.003 mmol). Reaction time: 4 h. Purified by column chromatography (eluent: hexane-EtOAc = 4:1) to yield 167 mg (85%) brown amorphous solid. R_f_ = 0.13 (EtOAc-hexane = 1:4); [α]_D_ = −2 (c 0.53, CHCl_3_); ^1^H-NMR (CDCl_3_, 360 MHz) δ (ppm): 7.92–7.89 (2H, m, Ar), 7.86 (1H, s, triazole H-5), 7.55-7.07 (25H, m, Ar), 4.99, 4.95 (2 × 1H, 2 d, *J* = 11.1 Hz, PhC*H*_2_), 4.89, 4.61 (2 × 1H, 2 d, *J* = 10.7 Hz, PhC*H*_2_), 4.79, 4.49 (2 × 1H, 2 d, *J* = 10.7 Hz, PhC*H*_2_), 4.59, 4.54 (2 × 1H, 2 d, *J* = 12.2 Hz, PhC*H*_2_), 4.70 (1H, d, *J* = 9.8 Hz, H-1’), 4.16, 3.90 (2H, 2 pseudo t, *J* = 9.4, 8.9 Hz, H-2’ and/or H-3’ and/or H-4’), 3.83–3.70 (4H, m, H-2’ or H-3’ or H-4’, H-5’, H-6’a, H-6’b); ^13^C-NMR (CDCl_3_, 90 MHz) δ (ppm): 145.2 (triazole C-4), 138.4–122.1 (Ar), 125.7 (triazole C-5), 86.4, 81.6, 79.4, 78.1, 73.8 (C-1’–C-5’), 75.5, 75.0, 74.9, 73.3 (4 × Ph*C*H_2_), 69.0 (C-6’). ESI-HRMS positive mode (*m*/*z*): calcd. for C_46_H_44_N_3_O_5_^+^ ([M + H]^+^): 718.3275. Found: 718.3270.

*1-(Naphthalen-2-yl)-4-(2’,3’,4’,6’-tetra-O-acetyl-β-d-glucopyranosyl)-1H-1,2,3-triazole* (**4b**). Method A: To the solution of **2b** (106 mg, 0.15 mmol) in anhydr. CH_2_Cl_2_ (4 mL) and acetic anhydride (4 mL) trimethylsilyl trifluoromethanesulfonate (214 μL, 1.18 mmol) was added at −40 °C. The mixture was slowly allowed to warm up and stirred at r.t. for 24 h, then at 50 °C for 24 h. Saturated aqueous NaHCO_3_ (2 mL) was added to the reaction mixture at 0 °C and the mixture was extracted with CH_2_Cl_2_ (3 × 5 mL). The combined organic phases were dried, concentrated and purified by column chromatography (hexane-EtOAc 2:1) to yield 53 mg (68%) product.

Method B: Prepared according to general procedure 2 from 2-naphthylboronic acid (80 mg, 0.47 mmol), CuSO_4_·5H_2_O (12 mg, 0.05 mmol), NaN_3_ (36 mg, 0.56 mmol), L-ascorbic acid (41 mg, 0.23 mmol) and **3** (50 mg, 0.14 mmol). Reaction time: 1.5 h. Purified by column chromatography (eluent: hexane-CH_2_Cl_2_-EtOAc 5:4:1) to yield 59 mg (80%) product.

White crystals. R_f_ = 0.31 (hexane-EtOAc 1:1); Mp: 225–227 °C; [α]_D_ = −71 (c 0.54, CHCl_3_); ^1^H-NMR (CDCl_3_, 360 MHz) δ (ppm): 8.19 (1H, s, triazole H-5), 8.16 (1H, s, Ar), 8.01–7.85 (4H, m, Ar), 7.59–7.57 (2H, m, Ar), 5.45–5.38 (2H, m, H-2’ and/or H-3’ and/or H-4’), 5.23 (1H, pseudo t, *J* = 9.7, 9.5 Hz, H-2’ or H-3’ or H-4’), 4.90 (1H, d, *J* = 9.6 Hz, H-1’), 4.33 (1H, dd, *J* = 12.4, 4.9 Hz, H-6’a), 4.17 (1H, dd, *J* = 12.4, 1.4 Hz, H-6’b), 3.94 (1H, ddd, *J* = 9.9, 4.7, 1.6 Hz, H-5’), 2.09, 2.08, 2.04, 1.96 (4 × 3H, 4 s, C*H*_3_CO); ^13^C-NMR (CDCl_3_, 90 MHz) δ (ppm): 170.6, 170.1, 169.6, 169.5 (CH_3_*C*O), 144.9 (C-4), 134.1, 133.1, 132.9, 129.9, 128.2, 127.9, 127.5, 127.0, 118.8 (Ar), 120.6 (C-5), 76.3, 73.9, 73.2, 71.2, 68.4 (C-1’–C-5’), 62.1 (C-6’), 20.7 (*C*H_3_CO), 20.6 (3 × *C*H_3_CO). ESI-HRMS positive mode (*m*/*z*): calcd. for C_26_H_27_N_3_NaO_9_^+^ ([M + Na]^+^ ): 548.1640. Found: 548.1636.

*1-(Naphthalen-1-yl)-4-(2’,3’,4’,6’-tetra-O-acetyl-β-d-glucopyranosyl)-1H-1,2,3-triazole* (**4c**) and *1-(5,6,7,8-tetrahydronaphthalen-1-yl)-4-(2’,3’,4’,6’-tetra-O-acetyl-β-d-glucopyranosyl)-1H-1,2,3-triazole* (**4d**)

Method A: To the solution of 1-(naphthalen-1-yl)-4-(2’,3’,4’,6’-tetra-*O*-benzyl-β-d-glucopyranosyl)-1*H*-1,2,3-triazole (**2c**, 159 mg, 0.22 mmol) in anhydr. EtOAc (5 mL) Pd(C) (10 wt. %, 16 mg) was added and the mixture was stirred in H_2_ atmosphere (1 bar) at 40 °C for 5 h. The catalyst was removed by filtration and the filtrate was concentrated and the residue was dissolved in pyridine (2.5 mL) and acetic anhydride (1 mL) and kept at 90 °C for 3 h. After evaporation the products were separated by column chromatography (hexane-EtOAc 2:1) to yield **4c** (34 mg, 29%) and **4d** (3 mg, 3%).

Method B: Compound **4c** was prepared according to general procedure 1 from alkyne **3** (50 mg, 0.14 mmol), 1-azidonaphthalene (15 mg, 0.14 mmol) and CuO(CO)C_3_H_7_(PPh_3_)_2_ (0.6 mg, 0.001 mmol). Reaction time: 5 h. Isolation by column chromatography (hexane-EtOAc 2:1 → 1:1 gradient) yielded **4c** (67 mg, 91%).

**4c**: white crystals. R_f_ = 0.30 (hexane-EtOAc = 1:1); Mp: 195–197 °C; [α]_D_ = −29 (c 0.5, CHCl_3_); ^1^H-NMR (CDCl_3_, 360 MHz) δ (ppm): 8.00 (1H, s, triazole H-5), 8.04–7.97 (2H, m, Ar), 7.60–7.55 (5H, m, Ar), 5.47–5.38 (2H, m, H-2’ and/or H-3’ and/or H-4’), 5.23 (1H, pseudo t, *J* = 9.7, 9.3 Hz, H-2’ or H-3’ or H-4’), 4.94 (1H, d, *J* = 9.4 Hz, H-1’), 4.32 (1H, dd, *J* = 12.5, 4.8 Hz, H-6’a), 4.18 (1H, dd, *J* = 12.5, 1.8 Hz, H-6’b), 3.95 (1H, ddd, *J* = 9.9, 4.7, 1.8 Hz, H-5’), 2.09, 2.07, 2.04, 1.99 (4 × 3H, 4 s, C*H*_3_CO); ^13^C-NMR (CDCl_3_, 90 MHz) δ (ppm): 170.6, 170.1, 169.6, 169.5 (CH_3_*C*O), 144.1 (triazole C-4), 134.1, 133.4, 130.5, 128.4, 128.2, 127.9, 127.0, 124.9, 123.6, 122.1 (Ar), 125.0 (triazole C-5), 76.3, 73.9, 73.4, 71.4, 68.4 (C-1’–C-5’), 62.1 (C-6’), 20.7 (*C*H_3_CO), 20.6 (3 × *C*H_3_CO). ESI-HRMS positive mode (*m*/*z*): calcd. for C_26_H_27_N_3_NaO_9_^+^ ([M + Na]^+^): 548.1640. Found: 548.1639.

**4d**: colouress syrup. R_f_ = 0.40 (hexane-EtOAc 1:1); [α]_D_ = −49 (c 0.15, CHCl_3_); ^1^H-NMR (CDCl_3_, 360 MHz) δ (ppm): 7.75 (1H, s, H-5), 7.26–7.22 (2H, m, Ar), 7.12 (1H, m, Ar), 5.42–5.33 (2H, m, H-2’ and/or H-3’ and/or H-4’), 5.21 (1H, pseudo t, *J* = 9.6, 9.4 Hz, H-2’ or H-3’ or H-4’), 4.86 (1H, d, *J* = 9.4 Hz, H-1’), 4.30 (1H, dd, *J* = 12.4, 4.8 Hz, H-6’a), 4.16 (1H, dd, *J* = 12.5, <1 Hz, H-6’b), 3.92 (1H, ddd, *J* = 10.0, 4.6, 1.9 Hz, H-5’), 2.86 (2H, pseudo t, *J* = 5.8, 6.2 Hz, tetralin H-5’’), 2.40 (2H, pseudo t, *J* = 5.6, 6.2 Hz, tetralin H-8’’), 2.08, 2.07, 2.02, 1.93 (4 × 3H, 4 × s, C*H*_3_CO), 1.82–1.70 (4H, m, tetralin H-6’’, H-7’’). ESI-HRMS positive mode (*m*/*z*): calcd. for C_26_H_31_N_3_NaO_9_^+^ ([M + Na]^+^): 552.1953. Found: 552.1953.

*1-Phenyl-4-(β-d-glucopyranosyl)-1H-1,2,3-triazole* (**5a**). Triazole **2a** (137 mg, 0.21 mmol) was dissolved in a 1:1 mixture of anhydr. EtOH and EtOAc (4 mL), Pd(C) (10 wt. %, 13 mg) was added and the mixture was stirred in H_2_ atmosphere (1 bar) at rt for 72 h. The catalyst was removed by filtration and the filtrate was purified by column chromatography (eluent: CHCl_3_-MeOH 7:3) to yield 58 mg (92%) colorless syrup. R_f_ = 0.46 (CHCl_3_-MeOH 7:3); [α]_D_ = +15 (c 1.14, MeOH); ^1^H-NMR (D_2_O, 360 MHz) δ (ppm): 8.30 (1H, s, triazole H-5), 7.50–7.48 (2H, m, Ar), 7.40–7.36 (3H, m, Ar), 4.55 (1H, d, *J* = 9.7 Hz, H-1’), 3.91 (1H, dd, *J* = 12.1, <1 Hz, H-6’a), 3.78–3.71 (2H, m, H-2’ or H-3’ or H-4’, H-6’b), 3.64 (1H, pseudo t, *J* = 8.8, 8.2 Hz, H-2’ or H-3’ or H-4’), 3.58–3.55 (2H, m, H-2’ or H-3’ or H-4’, H-5’); ^13^C-NMR (D_2_O, 90 MHz) δ (ppm): 145.6 (triazole C-4), 136.0, 129.9, 129.6, 120.8 (Ar), 123.1 (triazole C-5), 80.3, 77.4, 73.7, 73.2, 69.8 (C-1’–C-5’), 61.1 (C-6’). ESI-HRMS positive mode (*m*/*z*): calcd. for C_14_H_17_N_3_NaO_5_^+^ ([M + Na]^+^): 330.1060. Found: 330.1058.

*1-(Naphthalen-2-yl)-4-(β-d-glucopyranosyl)-1H-1,2,3-triazole* (**5b**). Prepared from compound **4b** (72 mg, 0.14 mmol) according to General procedure 3. Yield: 47 mg (96%) white crystals. R_f_ = 0.35 (CHCl_3_-MeOH 8:2); Mp: 224–225 °C; [α]_D_ = +12 (c 0.65, MeOH); ^1^H-NMR (DMSO-*d*_6_, 360 MHz) δ (ppm): 8.92 (1H, s, Ar), 8.45 (1H, s, triazole H-5), 8.17–8.01 (4H, m, Ar), 7.62 (2H, m, Ar), 4.36 (1H, d, *J* = 9.7 Hz, H-1’), 3.70 (1H, dd, *J* = 11.6, <1 Hz, H-6’a), 3.59 (1H, dd, *J* = 11.2, <1 Hz, H-6’b), 3.44 (1H, m, H-5’), 3.36–3.31 (2H, m, H-2’ and/or H-3’ and/or H-4’), 3.20 (1H, pseudo t, *J* = 9.0, 8.9 Hz; H-2’ or H-3’ or H-4’); ^13^C-NMR (DMSO-*d*_6_, 90 MHz) δ (ppm): 147.3 (triazole C-4), 134.3, 133.0, 132.4, 130.2, 128.4, 128.0, 127.7, 127.2, 122.3, 118.7, 117.8 (Ar), 81.4, 78.0, 74.3, 73.1, 70.3 (C-1’–C-5’), 61.3 (C-6’). ESI-HRMS positive mode (*m*/*z*): calcd. for C_18_H_19_N_3_NaO_5_^+^ ([M + Na]^+^): 380.1217. Found: 380.1216.

*1-(Naphthalen-1-yl)-4-(β-d-glucopyranosyl)-1H-1,2,3-triazole* (**5c**). Prepared from compound **4c** (79 mg, 0.15 mmol) according to General procedure 3. Yield: 51 mg (94%) pale brown syrup. R_f_ = 0.37 (CHCl_3_-MeOH 4:1); [α]_D_ = +7 (c 0.42, MeOH); ^1^H-NMR (CD_3_OD, 360 MHz) δ (ppm): 8.37 (1H, s, triazole H-5), 8.10–8.08 (1H, m, Ar), 8.02–8.00 (1H, m, Ar), 7.62–7.54 (1H, m, Ar), 4.59 (1H, d, *J* = 9.7 Hz, H-1’), 3.93 (1H, dd, *J* = 11.8, <1 Hz, H-6’a), 3.77–3.72 (2H, m, H-2’ and/or H-3’ and/or H-4’ and/or H-5’ and/or H-6’b), 3.62–3.50 (3H, m, H-2’ and/or H-3’ and/or H-4’ and/or H-5’ and/or H-6’b); ^13^C-NMR (CD_3_OD, 90 MHz) δ (ppm): 147.6 (triazole C-4), 135.6, 134.8, 131.8, 129.8, 129.5, 129.0, 128.3, 126.2, 124.9, 123.2 (Ar), 127.5 (triazole C-5), 82.4, 79.6, 75.7, 75.1, 71.6 (C-1’–C-5’), 63.0 (C-6’). ESI-HRMS positive mode (*m*/*z*): calcd. for C_18_H_19_N_3_NaO_5_^+^ ([M + Na]^+^): 380.1217. Found: 380.1216.

*1-(2’,3’,4’,6’-Tetra-O-acetyl-β-d-glucopyranosyl)-4-phenyl-1H-imidazole* (**7**). To a solution of 2,3,4,6-tetra-*O*-acetyl-α-d-glucopyranosyl-bromide (**6**, 0.20 g, 0.49 mmol) and 4-phenylimidazole (0.14 g, 0.97 mmol) in anhydr. CH_3_CN (5 mL) mercury(II) cyanide (0.12 g, 0.49 mmol) and activated 4 Å molecular sieves (powder, 200 mg) were added. The reaction mixture was heated at 60 °C until the TLC (hexane-EtOAc 2:3) showed disappearance of **6**. After cooling the reaction mixture to rt the insoluble inorganic salts and molecular sieves were filtered off, and the solution was evaporated under diminished pressure. The residue was dissolved in CHCl_3_ (30 mL) and extracted with 1M aq. KBr solution (2 × 20 mL) and water (20 mL), respectively. The organic layer was dried, filtered and evaporated. The residue was purified by column chromatography (hexane-EtOAc 2:3) to yield 140 mg (61%) white solid. M_p_ = 179–181 °C; R_f_ = 0.29 (hexane-EtOAc 1:2); [α]_D_ = −54 (c 0.22, CHCl_3_); ^1^H-NMR (400 MHz, CDCl_3_) δ (ppm): 7.77 (2H, dd, *J* = 7.2, 1.3 Hz, Ph), 7.67 (1H, d, *J* = 1.1 Hz, imidazole C*H*), 7.40–7.36 (3H, m, Ph, imidazole C*H*), 7.26 (1H, dt, *J* = 7.2, 1.4 Hz, Ph), 5.42, 5.37 (2 × 1H, 2 pseudo t, *J* = 9.2, 9.1 Hz in each, H-2’, H-3’), 5.34 (1H, d, *J* = 9.1 Hz, H-1’), 5.26 (1H, pseudo t, *J* = 9.9, 9.1 Hz, H-4’), 4.30 (1H, dd, *J* = 12.6, 5.0 Hz, H-6’a), 4.15 (1H, dd, *J* = 12.6, 2.1 Hz, H-6’b), 3.95 (1H, ddd, *J* = 9.9, 5.0, 2.1 Hz, H-5’), 2.09, 2.07, 2.03, 1.88 (4 × 3H, 4 s, 4 × C*H*_3_CO); ^13^C-NMR (100 MHz, CDCl_3_) δ (ppm): 170.6, 170.1, 169.4, 168.8 (4 × CH_3_*C*O), 143.2 (imidazole C-4), 136.9 (imidazole C-2), 133.5, 128.7 (2), 127.3, 125.1 (2) (Ph), 112.3 (imidazole C-5), 83.8 (C-1’), 75.0 (C-5’), 73.0, 70.6 (C-2’, C-3’), 67.9 (C-4’), 61.8 (C-6’), 20.8, 20.6 (2), 20.3 (4 × *C*H_3_CO). ESI-HRMS positive mode (*m*/*z*): calcd. for C_23_H_27_N_2_O_9_^+^ ([M + H]^+^): 475.1711. Found: 475.1711.

*1-(β-d-Glucopyranosyl)-4-phenyl-1H-imidazole* (**8**). Prepared from compound **7** (250 mg, 0.53 mmol) according to general procedure 3. Purification by recrystallisation from MeOH yielded 120 mg (75%) white solid. R_f_ = 0.45 (CHCl_3_-MeOH 7:3); M_p_ = 273–274 °C; [α]_D_ = +56 (c 0.22, DMSO); ^1^H-NMR (360 MHz, DMSO-*d*_6_ + 1 drop of D_2_O) δ (ppm): 7.84 (1H, s, imidazole *C*H), 7.77–7.73 (3H, m, Ph, imidazole *C*H), 7.35 (2H, t, *J* = 7.4 Hz, Ph), 7.20 (1H, t, *J* = 7.4 Hz, Ph), 5.13 (1H, d, *J* = 9.1 Hz, H-1’), 3.67 (1H, H-6’a), 3.54 (1H, pseudo t, *J* = 9.8, 9.1 Hz, H-2’ or H-3’ or H-4’), 3.46 (1H, dd, *J* = 11.6, 5.6 Hz, H-6’b), 3.40–3.31 (2H, m, H-2’ or H-3’ or H-4’, H-5’), 3.23 (1H, pseudo t, *J* = 9.1, 9.1 Hz, H-2’ or H-3’ or H-4’); ^-13^C-NMR (90 MHz, DMSO-*d*_6_) δ (ppm): 140.3 (imidazole C-4), 137.4 (imidazole C-2), 134.4, 128.3 (2), 126.1, 124.1 (2) (Ph), 114.1 (imidazole C-5), 85.4 (C-1’), 79.6, 77.0, 72.4, 69.6 (C-2’–C-5’), 60.8 (C-6’). ESI-HRMS positive mode (*m*/*z*): calcd. for C_15_H_21_N_2_O_5_^+^ ([M + H]^+^): 307.1288. Found: 307.1286.

*2-Phenyl-5-(2’,3’,4’,6’-tetra-O-benzoyl-β-d-glucopyranosyl)-2H-tetrazole* (**10a**)

Method A: To the solution of tosylhydrazone **12** (100 mg, 0.13 mmol) in anhydr. pyridine benzenediazonium tetrafluoroborate (25 mg, 0.13 mmol) was added at −40 °C. The mixture was allowed to reach −10 °C in 20 min then CH_2_Cl_2_ was added and the mixture was washed with 10% aqueous HCl, saturated aqueous NaHCO_3_ and brine. The organic phase was dried, evaporated and the residue was purified by column chromatography (eluent: hexane-EtOAc 1:4 → 1:3 gradient) to give 57 mg (61%) yellowish syrup.

Method B: To the solution of tetrazole **9** (300 mg, 0.46 mmol) in anhydr. CH_2_Cl_2_ (6 mL) phenylboronic acid (1.6 equiv., 90 mg, 0.74 mmol), CuCl_2_ (0.12 equiv., 7 mg, 0.06 mmol), *N*,*N*,*N*’,*N*’-tetra-methylethylenediamine (0.12 equiv., 8 µL, 0.06 mmol) and K_2_CO_3_ (1.1 equiv., 70 mg, 0.51 mmol) were added and the mixture was stirred at r.t. under air. After 20 h CH_2_Cl_2_ was added and the solution was washed with 10% aqueous NH_3_, water and brine. The organic phase was dried, evaporated and the residue was purified by column chromatography (eluent: hexane-EtOAc 1:4 → 1:3 gradient) to give 319 mg (95%) colorless syrup.

R_f_ = 0.43 (hexane-EtOAc 3:2); [α]_D_ = −5 (c 0.36, CHCl_3_); ^1^H-NMR (CDCl_3_, 400 MHz) δ (ppm): 8.03–7.80 (10H, m, Ar), 7.53–7.27 (15H, m, Ar), 6.23 (1H, pseudo t, *J* = 9.7, 9.8 Hz, H-2’ or H-3’ or H-4’), 6.09 (1H, pseudo t, *J* = 9.5, 9.5 Hz, H-2’ or H-3’ or H-4’), 5.90 (1H, pseudo t, *J* = 9.7, 9.8 Hz, H-2’ or H-3’ or H-4’), 5.38 (1H, d, *J* = 10.0 Hz, H-1’), 4.69 (1H, dd, *J* = 12.4, 2.9 Hz, H-6’a), 4.56 (1H, dd, *J* = 12.4, 5.1 Hz, H-6’b), 4.42 (1H, ddd, *J* = 9.9, 5.1, 2.9 Hz, H-5’); ^13^C-NMR (CDCl_3_, 90 MHz) δ (ppm): 166.1, 165.8, 165.1, 164.6 (Ph*C*O), 162.2 (tetrazole C-5), 136.6 (phenyl C-1”), 133.4-128.2 (Ar), 120.0 (phenyl C-2”), 77.0, 74.3, 72.5, 71.1, 69.4 (C-1’–C-5’), 63.2 (C-6’). ESI-HRMS positive mode (*m*/*z*): calcd. for C_41_H_32_N_4_NaO_9_^+^ ([M + Na]^+^): 747.2061. Found: 747.2060.

*2-Methyl-5-(2’,3’,4’,6’-tetra-O-benzoyl-β-d-glucopyranosyl)-2H-tetrazole* (**10e**) and *1-methyl-5-(2’,3’,4’,6’-tetra-O-benzoyl-β-d-glucopyranosyl)-1H-tetrazole* (**11e**). A solution of diazomethane was prepared by a portionwise addition of *N*-nitroso-*N*-methylurea (400 mg, 3.88 mmol) to a stirred mixture of diethyl ether (5 mL) and 40% *w*/*w* aqueous solution of KOH (5 mL) at 0 °C. Ethereal phase was added dropwise to the solution of tetrazole **9** (500 mg, 0.77 mmol) in anhydr. CH_2_Cl_2_ (22 mL) at r.t. After disappearance of the tetrazole (TLC, PhMe-EtOAc 4:1) the solvent was removed in vacuo, and the residue was purified by column chromatography (eluent: PhMe-EtOAc 20:1 → 10:1 gradient) to give **10e** (192 mg, 38%) and **11e** (194 mg, 38%).

**10e**: white amorphous solid. R_f_ = 0.46 (PhMe-EtOAc 4:1); [α]_D_ = +36 (c 0.45, CHCl_3_); ^1^H-NMR (CDCl_3_, 400 MHz) δ (ppm): 8.01–7.79 (8H, m, Ar), 7.50–7.21 (12H, m, Ar), 6.17–6.08 (2H, m, H-2’, H-3’), 5.90 (1H, pseudo t, *J* = 9.5, 9.4 Hz, H-4’), 5.35 (1H, d, *J* = 9.3 Hz, H-1’), 4.68 (1H, dd, *J* = 12.4, 2.9 Hz, H-6’a), 4.56 (1H, dd, *J* = 12.4, 5.1 Hz, H-6’b), 4.42 (1H, ddd, *J* = 9.6, 5.0, 2.8 Hz, H-5’), 4.19 (3H, s, C*H*_3_); ^13^C-NMR (CDCl_3_, 90 MHz) δ (ppm): 166.0, 165.7, 165.0, 164.4 (Ph*C*O), 162.1 (tetrazole C-5), 133.3–128.2 (Ar), 76.8, 74.2, 72.4, 71.1, 69.3 (C-1’–C-5’), 63.1 (C-6’), 39.4 (*C*H_3_). ESI-HRMS positive mode (*m*/*z*): calcd. for C_36_H_30_N_4_NaO_9_^+^ ([M + Na]^+^): 685.1905. Found: 685.1900.

**11e**: white amorphous solid. R_f_ = 0.34 (PhMe-EtOAc 4:1); [α]_D_ = −7 (c 0.53, CHCl_3_); ^1^H-NMR (CDCl_3_, 400 MHz) δ (ppm): 8.05–7.77 (8H, m, Ar), 7.58–7.23 (12H, m, Ar), 6.19 (1H, pseudo t, *J* = 9.6, 9.6 Hz, H-3’), 5.90 (1H, pseudo t, *J* = 9.8, 9.9 Hz, H-4’), 5.83 (1H, pseudo t, *J* = 9.9, 9.9 Hz, H-2’), 5.49 (1H, d, *J* = 10.2 Hz, H-1’), 4.74 (1H, dd, *J* = 12.5, 2.6 Hz, H-6’a), 4.54 (1H, dd, *J* = 12.5, 4.9 Hz, H-6’b), 4.42 (1H, ddd, *J* = 9.3, 4.7, 2.6 Hz, H-5’), 4.24 (3H, s, C*H*_3_); ^13^C-NMR (CDCl_3_, 90 MHz) δ (ppm): 165.8, 165.5, 165.1 (2) (Ph*C*O), 149.9 (tetrazole C-5), 133.6–127.9 (Ar), 77.2, 73.1, 71.8, 69.8, 68.8 (C-1’–C-5’), 62.3 (C-6’), 34.7 (*C*H_3_). ESI-HRMS positive mode (*m*/*z*): calcd. for C_36_H_30_N_4_NaO_9_^+^ ([M + Na]^+^): 685.1905. Found: 685.1902.

*2-Phenyl-5-(β-d-glucopyranosyl)-2H-tetrazole* (**13a**). Prepared from compound **10a** (200 mg, 0.28 mmol) according to general procedure 3. Yield: 80 mg (94%) white amorphous solid. R_f_ = 0.41 (CHCl_3_-MeOH 4:1); [α]_D_ = +12 (c 0.27, MeOH); ^1^H-NMR (DMSO-d_6_, 360 MHz) δ (ppm): 8.10–8.07 (2H, m, Ar), 7.70–7.59 (3H, m, Ar), 4.58 (1H, d, *J* = 9.8 Hz, H-1’), 3.77–3.69 (2H, m, H-2’ and/or H-3’ and/or H-4’ and/or H-5’ and/or H-6’), 3.48–3.34 (3H, m, H-2’ and/or H-3’ and/or H-4’ and/or H-5’ and/or H-6’), 3.23 (1H, pseudo t, *J* = 9.1, 9.0 Hz, H-2’ or H-3’ or H-4’); ^13^C-NMR (DMSO-d_6_, 90 MHz) δ (ppm): 164.8 (tetrazole C-5), 136.2, 130.3, 130.2, 119.9 (Ar), 81.7, 77.6, 73.4, 72.5, 70.1 (C-1’–C-5’), 61.1 (C-6’). ESI-HRMS positive mode (*m*/*z*): calcd. for C_13_H_16_N_4_NaO_5_^+^ ([M + Na]^+^): 331.1013. Found: 331.1013.

*2-Methyl-5-(β-d-glucopyranosyl)-2H-tetrazole* (**13e**). Prepared from compound **10e** (150 mg, 0.23 mmol) according to general procedure 3. Yield: 40 mg (72%) colourless syrup. R_f_ = 0.41 (CHCl_3_-MeOH 7:3); [α]_D_ = +1 (c 0.63, MeOH); ^1^H-NMR (D_2_O, 400 MHz) δ (ppm): 4.76 (1H, d, *J* = 10.0 Hz, H-1’), 4.41 (3H, s, C*H*_3_), 3.92 (1H, dd, *J* = 12.4, 1.8 Hz, H-6’a), 3.81 (1H, pseudo t, *J* = 9.3, 9.7 Hz, H-2’ or H-3’ or H-4’), 3.75 (1H, dd, *J* = 12.4, 5.7 Hz, H-6’b), 3.68-3.58 (2H, m, H-2’ or H-3’ or H-4’, H-5’), 3.56 (1H, pseudo t, *J* = 9.2, 9.4 Hz, H-2’ or H-3’ or H-4’); ^13^C-NMR (D_2_O, 100 MHz) δ (ppm): 163.9 (tetrazole C-5), 81.0, 77.5, 73.6, 73.1, 70.1 (C-1’–C-5’), 61.4 (C-6’), 40.4 (*C*H_3_). ESI-HRMS positive mode (*m*/*z*): calcd. for C_8_H_14_N_4_NaO_5_^+^ ([M + Na]^+^): 269.0856. Found: 269.0855.

*1-Methyl-5-(β-d-glucopyranosyl)-1H-tetrazole* (**14e**). Prepared from compound **11e** (150 mg, 0.23 mmol) according to general procedure 3. Yield: 54 mg (97%) pale yellow syrup. R_f_ = 0.33 (CHCl_3_-MeOH 7:3); [α]_D_ = +7 (c 0.83, MeOH); ^1^H-NMR (D_2_O, 360 MHz) δ (ppm): 4.91 (1H, d, *J* = 9.7 Hz, H-1’), 4.17 (3H, s, C*H*_3_), 3.92 (1H, dd, *J* = 12.4, <1 Hz, H-6’a), 3.83–3.66 (4H, m, H-2’ and/or H-3’ and/or H-4’ and/or H-5’ and/or H-6’b), 3.57 (1H, pseudo t, *J* = 9.4, 9.1 Hz, H-2’ or H-3’ or H-4’); ^13^C-NMR (D_2_O, 90 MHz) δ (ppm): 153.9 (tetrazole C-5), 81.1, 77.3, 73.0, 71.5, 69.9 (C-1’–C-5’), 61.4 (C-6’), 35.1 (*C*H_3_). ESI-HRMS positive mode (*m*/*z*): calcd. for C_8_H_14_N_4_NaO_5_^+^ ([M + Na]^+^): 269.0856. Found: 269.0857.

*5-Phenyl-2-(2’,3’,4’,6’-tetra-O-acetyl-β-d-glucopyranosyl)-2H-tetrazole* (**15a**) and *5-phenyl-1-(2’,3’,4’,6’-tetra-O-acetyl-β-d-glucopyranosyl)-1H-tetrazole* (**16a**). Prepared according to general procedure 4 from bromide **6** (500 mg, 1.22 mmol) and 5-phenyltetrazole (355 mg, 2.43 mmol). Products were separated by column chromatography (eluent: hexane-acetone 3:1) to give **15a** (460 mg, 79%) and **16a** (96 mg, 17%). ^1^H and ^13^C-NMR spectra of the isolated compounds are in agreement with those reported earlier [61].

*5-Methyl-2-(2,3,4,6-tetra-O-acetyl-β-d-glucopyranosyl)-2H-tetrazole (***15e**). Prepared according to general procedure 4 from bromide **6** (500 mg, 1.22 mmol) and 5-methyltetrazole (204 mg, 2.43 mmol). Products were separated by column chromatography (eluent: hexane-acetone 9:1 → 4:1 gradient) to give **15e** (131 mg, 26%) and glycal **17** [67] (181 mg, 45%).

**15e**: White amorphous solid. R_f_ = 0.48 (hexane-acetone 1:1); [α]_D_ = +7 (c 0.46, CHCl_3_); ^1^H-NMR (CDCl_3_, 400 MHz) δ (ppm): 6.06 (1H, d, *J* = 9.4 Hz, H-1’), 5.83 (1H, pseudo t, *J* = 9.4, 9.4 Hz, H-2’ or H-3’ or H-4’), 5.42 (1H, pseudo t, *J* = 9.5, 9.5 Hz, H-2’ or H-3’ or H-4’), 5.30 (1H, pseudo t, *J* = 9.9, 9.7 Hz, H-2’ or H-3’ or H-4’), 4.30 (1H, dd, *J* = 12.7, 5.0 Hz, H-6’a), 4.17 (1H, dd, *J* = 12.7, 2.0 Hz, H-6’b), 4.03 (1H, ddd, *J* = 10.0, 5.0, 2.0 Hz, H-5’), 2.58 (3H, s, C*H*_3_), 2.08, 2.08, 2.04, 1.85 (4 × 3H, s, 4 × C*H*_3_CO); ^13^C-NMR (CDCl_3_, 100 MHz) δ (ppm): 170.6, 170.2, 169.2, 168.4 (CH_3_*C*O), 163.9 (tetrazole C-5), 86.5 (C-1’), 75.1, 73.0, 69.8, 67.4 (C-2’–C-5’), 61.5 (C-6’), 20.7, 20.6 (2), 20.2 (*C*H_3_CO), 11.1 (*C*H_3_). ESI-HRMS positive mode (*m*/*z*): calcd. for C_16_H_22_N_4_NaO_9_^+^ ([M + Na]^+^): 437.1279. Found: 437.1281.

*5-Phenyl-2-(β-d-glucopyranosyl)-2H-tetrazole* (**18a**). Prepared from compound **15a** (155 mg, 0.33 mmol) according to General procedure 3. Yield: 85 mg (85%) white amorphous solid. R_f_ = 0.29 (CHCl_3_-MeOH 4:1); [α]_D_ = −4 (c 1.31, MeOH); ^1^H-NMR (D_2_O, 400 MHz) δ (ppm): 8.01-7.98 (2H, m, Ar), 7.54–7.50 (3H, m, Ar), 6.08 (1H, d, *J* = 9.2 Hz, H-1’), 4.20 (1H, pseudo t, *J* = 9.3, 9.3 Hz, H-2’ or H-3’ or H-4’), 3.97–3.93 (1H, m, H-2’ or H-3’ or H-4’ or H-5’ or H-6’), 3.82–3.76 (3H, m, H-2’ and/or H-3’ and/or H-4’ and/or H-5’ and/or H-6’), 3.67 (1H, pseudo t, *J* = 9.2, 9.2 Hz, H-2’ or H-3’ or H-4’); ^13^C-NMR (D_2_O, 100 MHz) δ (ppm): 165.9 (tetrazole C-5), 131.9, 129.9, 127.5, 126.3 (Ar), 89.9 (C-1’), 79.7, 76.4, 72.5, 69.5 (C-2’–C-5’), 61.1 (C-6’). ESI-HRMS positive mode (*m*/*z*): calcd. for C_13_H_16_N_4_NaO_5_^+^ ([M + Na]^+^): 331.1013. Found: 331.1014.

*5-Methyl-2-(β-d-glucopyranosyl)-2H-tetrazole* (**18e**). Prepared from compound **15e** (136 mg, 0.33 mmol) according to general procedure 3. Yield: 68 mg (84%) white amorphous solid. R_f_ = 0.29 (CHCl_3_-MeOH 4:1); [α]_D_ = −14 (c 0.66, CHCl_3_); ^1^H-NMR (D_2_O, 400 MHz) δ (ppm): 6.03 (1H, d, *J* = 9.2 Hz, H-1’), 4.13 (1H, pseudo t, *J* = 9.2, 9.3 Hz, H-2’ or H-3’ or H-4’), 3.93 (1H, dd, *J* = 10.7, <1 Hz, H-6’a), 3.82–3.73 (2H, m, H-5’ and H-6’b), 3.75 (1H, pseudo t, *J* = 9.2, 9.3 Hz, H-2’ or H-3’ or H-4’), 3.64 (1H, pseudo t, *J* = 9.3, 9.3 Hz, H-2’ or H-3’ or H-4’), 2.58 (3H, s, C*H*_3_); ^13^C-NMR (D_2_O, 100 MHz) δ (ppm): 164.7 (tetrazole C-5), 89.5 (C-1’), 79.6, 76.4, 72.4, 69.5 (C-2’–C-5’), 61.0 (C-6’), 10.5 (*C*H_3_). ESI-HRMS positive mode (*m*/*z*): calcd. for C_8_H_14_N_4_NaO_5_^+^ ([M + Na]^+^): 269.0856. Found: 269.0858.

*5-Phenyl-1-(β-d-glucopyranosyl)-1H-tetrazole* (**19a**). Prepared from compound **16a** (86 mg, 0.18 mmol) according to general procedure 3. Yield: 48 mg (86%) colourless syrup. R_f_ = 0.29 (CHCl_3_-MeOH 4:1); [α]_D_ = +17 (c 0.68, MeOH); ^1^H-NMR (D_2_O, 400 MHz) δ (ppm): 7.74–7.67 (3H, m, Ar), 7.64–7.60 (2H, m, Ar), 5.60 (1H, d, *J* = 9.1 Hz, H-1’), 4.31 (1H, pseudo t, *J* = 9.1, 9.1 Hz, H-2’ or H-3’ or H-4’), 3.95 (1H, dd, *J* = 12.4, 1.7 Hz, H-6’a), 3.78 (1H, dd, *J* = 12.4, .6 Hz, H-6’b), 3.71, (1H, ddd, *J* = 9.4, 5.8, 1.4 Hz, H-5’), 3.66–3.62 (2H, m, H-2’ and/or H-3’ and/or H-4’); ^13^C-NMR (D_2_O, 100 MHz) δ (ppm): 157.3 (tetrazole C-5), 133.0, 130.1, 129.8, 122.3 (Ar), 85.5, 79.5, 76.5, 72.0, 69.6 (C-1’–C-5’), 61.1 (C-6’). ESI-HRMS positive mode (*m*/*z*): calcd. for C_13_H_16_N_4_NaO_5_^+^ ([M + Na]^+^): 331.1013. Found: 331.1012.

## 4. Conclusions

In this study, initiated by the objective of extending the structure-activity relationships of *C*- and *N*-β-d-glucopyranosyl derivatives of a wide range of azole type heterocycles as glycogen phosphorylase inhibitors, new methyl and aryl substituted 1,2,3-triazoles, imidazoles and tetrazoles have been synthesized. Enzyme kinetic investigation of the new compounds showed most of them to have no significant inhibitory activity against RMGP*b*. These and previous [44] experiences with β-d-Glc*_p_*-azole-Ar type compounds indicate that the inhibitory effect of these is highly sensible to the structure of the azole moieties and the range of efficacy expands from inactives to the best known glucose derived inhibitors. The understanding of such an enormous variability seems to be beyond simple or intuitive discretion, therefore, computational studies are underway to get a deeper insight in these phenomena. 

## Figures and Tables

**Table 1:** Selected glucose derived inhibitors of rabbit muscle glycogen phosphorylase *b* (*K*_i_ [μM]).

R					CH_3_		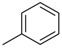		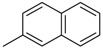	
					a		b		c	
**I**	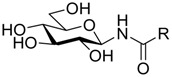				32 [26]		81 [26] 144 [27]		10 [28] 13 [29]	
**II**	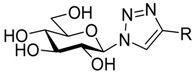				-		151 [29] 162 [34]		16 [29] 36 [34]	
**III**	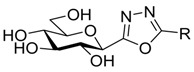				212 [35] 145 [36]		10% at 625 μM [37]		10% at 625 μM [37]	
**IV**	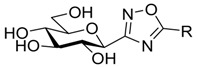				No inh. at 625 μM [38]		10% at 625 μM [38]		38 [38]	
**V**	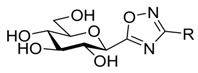				-		27 [39] 64 [37]		12 * [37]	
**VI**	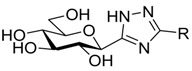				499 [41]		7 [40,41]		0.41 [40,41]	
**VII**	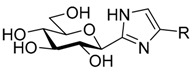				-		0.28 [42,43]		0.031 [42,43]	
**VIII**	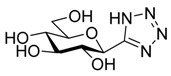				No inh. at 625 μM [35]					
* A *K*_i_ value of 2.4 μM was measured by N.G. Oikonomakos et al. (unpublished results in ref. [37])										
**Target compounds in this study:**						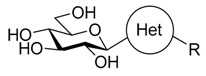				
**IX**		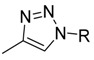	**X**	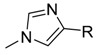		**XI**		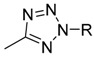	**XII**	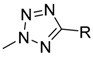

**Table 2:** Synthesis of 1-aryl-4-(β-d-glucopyranosyl)-1,2,3-triazoles.

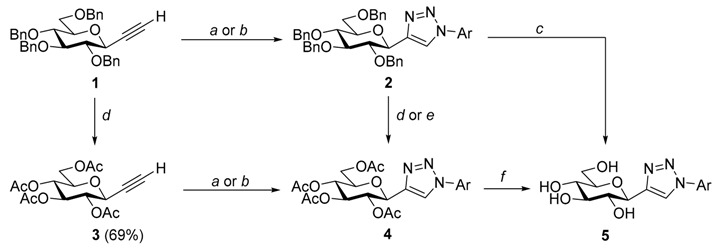							
Reagents and conditions: (***a***) ArN_3_, CuO(CO)C_3_H_7_(PPh_3_)_2_, dry CH_2_Cl_2_, r.t.; (***b***) і. ArB(OH)_2_, NaN_3_, CuSO_4_·5H_2_O, MeOH, r.t., іі. **1** or **3**, L-ascorbic acid, CH_2_Cl_2_-H_2_O (1:1), 50 °C; (***c***) H_2_, Pd(C), dry EtOH, dry EtOAc, r.t.; (***d***) TMSOTf, Ac_2_O, −40 °C; (***e***) і. H_2_, Pd(C), dry EtOAc, dry MeOH, 40 °C, іі. Ac_2_O, pyridine, 90 °C; (***f***) ~1M NaOMe in MeOH, r.t.							
**Ar**		**Conditions and Yields (%)**					
			**2**		**4**		**5**
**a**	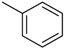	*a*	78 (from **1**)	-	-	*c*	92 (from **2a**)
**b**	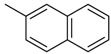	*b*	79 (from **1**)	*d*	68 (from **2b**)	*f*	96 (from **4b**)
				*b*	80 (from **3**)		
**c**	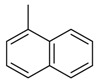	*a*	85 (from **1**)	*e*	29 (from **2c**)	*f*	94 (from **4c**)
				*a*	91 (from **3**)		
**d**	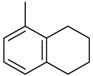	-	-	*e*	3 (from **2c**)	-	-

**Table 3:** Synthesis of 5-(β-d-glucopyranosyl)-*N*-substituted-tetrazoles.

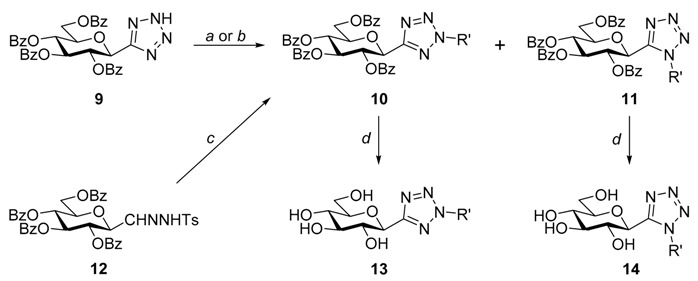											
Reagents and conditions: (***a***) R’B(OH)_2_, CuCl_2_, TMEDA, K_2_CO_3_, dry CH_2_Cl_2_, r.t.; (***b***) CH_2_N_2_ in Et_2_O, dry CH_2_Cl_2_, r.t.; (***c***) PhN_2_BF_4_, dry pyridine, −40 °C; (***d***) ~1M NaOMe in MeOH, r.t.											
		**Conditions, Yields (%) and *Chemical Shifts (ppm) for Tetrazole C-5* (Solvent)**									
**R’**			**10**		**11**			**13**		**14**	
**a**	Phenyl	*a*	95	***162.2*** (CDCl_3_)	-	-	*d*	94	***164.8*** (DMSO-*d*_6_)	-	-
		*c*	61		-						
**e**	Methyl	*b*	38	***162.1*** (CDCl_3_)	38	***149.9*** (CDCl_3_)	*d*	72	***163.9*** (D_2_O)	97	***153.9*** (D_2_O)

**Table 4:** Synthesis of *N*-(β-d-glucopyranosyl)-5-substituted-tetrazoles.

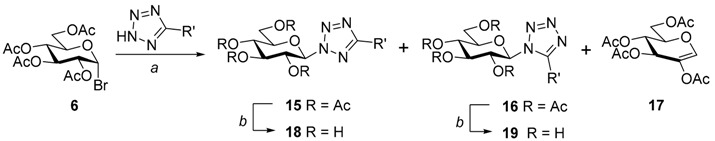												
Reagents and conditions: (***a***) K_2_CO_3_, 4 Å molecular sieves, dry acetone, reflux; (***b***) ~1 M NaOMe in MeOH, r.t.												
**R’**		**Conditions, Yields (%) and *Chemical Shifts (ppm) for Tetrazole C-5* (Solvent)**										
			**15**		**16**		**17**		**18**		**19**	
**a**	Phenyl	*a*	79	***165.8*** (CDCl_3_)	17	***155.9*** (CDCl_3_)	-	*b*	85	***165.9*** (D_2_O)	86	***157.3*** (D_2_O)
**e**	Methyl	*a*	26	***163.9*** (CDCl_3_)	-		45	*b*	84	***164.7*** (D_2_O)	-	

**Table 5:** Inhibitory effect of the new and some earlier compounds against rabbit muscle glycogen phosphorylase *b* (RMGP*b*).

Entry	Compound		Inhibition * (μM)
1.	**Ib**	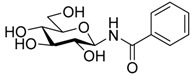	*K*_i_ 81 [26] *K*_i_ 144 [27]
2.	**IIb**	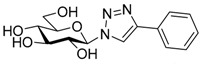	*K*_i_ 151 [29] *K*_i_ 162 [34]
3.	**20**	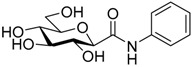	*K*_i_ 5400 [65]
4.	**5a**	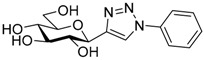	N.I.
5.	**5b**	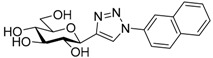	N.I.
6.	**5c**	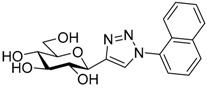	N.I.
7.	**8**	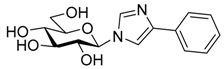	N.I.
8.	**13a**	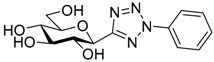	N.I.
9.	**13e**	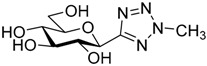	N.I.
10.	**14e**	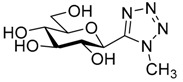	N.I.
11.	**18a**	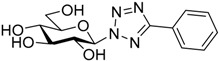	IC_50_ 600 (calculated ** *K*_i_ 327)
12.	**18e**	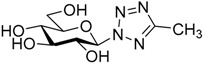	N.I.
13.	**19a**	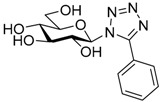	N.I.

* N.I. no inhibition at 625 μM concentration; ** Calculated by the Cheng-Prusoff equation [66].
